# Autaptic Connections and Synaptic Depression Constrain and Promote Gamma Oscillations

**DOI:** 10.1371/journal.pone.0089995

**Published:** 2014-02-28

**Authors:** William M. Connelly

**Affiliations:** Neuroscience Division, School of Biosciences, Cardiff University, Life Sciences Building, Cardiff, United Kingdom; University of Michigan, United States of America

## Abstract

Computational models of gamma oscillations have helped increase our understanding of the mechanisms that shape these 40–80 Hz cortical rhythms. Evidence suggests that interneurons known as basket cells are responsible for the generation of gamma oscillations. However, current models of gamma oscillations lack the dynamic short term synaptic plasticity seen at basket cell-basket cell synapses as well as the large autaptic synapses basket cells are known to express. Hence, I sought to extend the Wang-Buzsáki model of gamma oscillations to include these features. I found that autapses increased the synchrony of basket cell membrane potentials across the network during neocortical gamma oscillations as well as allowed the network to oscillate over a broader range of depolarizing drive. I also found that including realistic synaptic depression filtered the output of the network. Depression restricted the network to oscillate in the 60–80 Hz range rather than the 40–120 Hz range seen in the standard model. This work shows the importance of including accurate synapses in any future model of gamma oscillations.

## Introduction

Gamma oscillations are signals at 40–80 Hz that can be recorded in the hippocampus and neocortex [1]. They are believed to play a critical role in many higher order brain functions such as temporal encoding and feature binding [2], [3], [4], [5]. Numerous lines of evidence suggest that the activity of a specific class of interneuron, the basket cell, is responsible for the generation of gamma oscillations. Specifically, basket cells spiking correlates strongly with gamma activity [6]. Optogenetically generated activity in basket cells is sufficient to generate gamma oscillations, while inhibition of basket cells reduces gamma power [7]. Finally, computational models suggest that, under realistic physiological conditions, networks of coupled basket cells are minimal generators of gamma oscillations [8]. The most widely cited model of gamma oscillations is the Wang-Buzsáki (WB) model [9]. In this model, the reciprocal electrical and chemical synapses between basket cells are enough to generate 40-80 Hz oscillations in response to network depolarization. Subsequent modifications to bring it more in line with physiological reality have made this model more robust to inhomogeneity but also allowed it to oscillate over a 20–120 Hz range [10]. While this model is explicitly a model of hippocampal gamma, the basic underlying physiology of the hippocampal and neocortical basket cell system (convergences, divergence, IPSC kinetics etc.) are very similar, and hence there have been assumptions that neocortical gamma operates by a similar mechanism e.g. [7]. There is also a competing theory. As reviewed by Tiesinga and Sejnowski [11], the pyramidal-interneuron gamma (PING) model specifies that the excitatory drive has a larger effect on pyramidal neurons, which in turn activate local basket cells. These inhibitory cells feed back to pyramidal cells, inhibiting them for one gamma cycle. When the inhibition wanes, the pyramidal cells fire again. Thus, the PING model differs from the WB model, in that excitatory traffic into the system specifically targets pyramidal cells, rather than targeting interneurons. It is currently unclear which of these models likely represents the true mechanism in the neocortex, and potentially both are viable, depending on the physiological state of the cortex [11]. However, it seems that the WB model may more accurately represent the situation in the neocortex, as neocortical basket cells receive much stronger and more reliable glutamatergic thalamocortical excitation than pyramidal cells [12], [13]. However, there are limitations to the WB model and its subsequent modifications, most importantly that it does not include realistic synaptic depression and that it is lacking the autaptic connections that neocortical basket cells express [14], [15], [16]. The inclusion of autaptic synapses seems likely to affect the behavior of a neocortical oscillation for two reasons. Firstly, the autapses of neocortical basket cells are the highest conductance and most reliably formed synapses in the neocortex (85–90% of FS cells have measurable autaptic currents, with a mean conductance of 6–11 nS [14], [15] (cf. FS → FS synapses are formed in 60–80% of pairs, with a conductance of ∼1 nS [17], [18] FS → Pyramidal cell synapses are found in ∼60% of pairs, with a conductance of ∼3 nS [19], [20]). Secondly, autapses have already been demonstrated to improve the spike timing accuracy of single neurons [21]. Likewise, it seems probable that short term plasticity of synaptic strength will change the behavior of a model of gamma oscillations because short term plasticity in basket cells is very active at the frequencies of spiking found in models of gamma oscillations [8], [17]. Thus I sought to investigate the consequence of including autaptic connections and realistic synaptic depression in a neocortical model of gamma oscillations.

## Materials and Methods

A 500 ms long simulation of a network of basket cells was undertaken using NEURON 7.1 [22]. Basket cells were modeled using an anatomically simple model based on that of Bush and Sejnowski [23]. Neuronal somata were 30 µm in diameter and 30 µm long and had two primary dendrites (2.5 µm in diameter, 50 µm long), the ends of each being connected to two secondary dendrites (1.6 µm in diameter, 150 µm long). Axial resistivity was 100 Ω·cm and membranes had a specific capacitance of 1 µF/cm^2^. Each soma contained Hodgkin-Huxley type active conductances based on those of Wang and Buzsáki [9] (as described by Bartos et al., [10]) *K* (E_K_ = −90 mV, g_K_ = 0.09 S/cm^2^) and *Na* (E_Na_ = 55 mV, g_Na_ = 0.08 S/cm^2^) and a passive leak conductance *L* (E_L_ = −65 mV, g_L_ = 0.00015 S/cm^2^). Dendritic compartments were passive, with only *L* (E_L_ = −65 mV, g_L_ = 0.00015 S/cm^2^). The network architecture was based loosely on that of Bartos et al., [10]. The network consisted of 200 basket cells formed into a loop (i.e. that the 200^th^ neuron is “next” to the 1^st^ neuron) to remove edge effects. Measurements from cat visual cortex show that basket cells make contact with 33–58 other basket cells [24], so synaptic connectivity was established with a probability of 0.6 over the nearest 50 to 90 neurons (the maximum number of cells possibly contacted is defined as the inhibitory divergence) with a delay equivalent to conduction velocity of 0.25 m/s and an inter-cell distance of 50 µm <9 ms [10]. The peak synaptic conductance was randomly generated for each connection from a log-normal distribution with a mean amplitude of 1 nS and a coefficient of variation of 1 [18], [25]. It has been demonstrated that for very nearby interneurons, the probability of gap junction coupling is 0.6 and the average conductance is 1.7 nS, and this decays to a probability of 0.4 and a conductance of 0.7 nS when the inter-cell distance is 200 µM. Furthermore, there are no connections beyond this distance [26]. Anatomical studies estimate that in the neocortex, each basket cell is electrically coupled to less than 10 other basket cells [27]. Therefore I connected interneurons by gap junctions to a maximum of 8–12 other cells. Gap junctions were simulated between each cell and its two or four nearest neighboring somata with a probability of 0.6 and a conductance of 1.7 nS, its next two or four nearest neighbors with a probability of 0.5 and a conductance of 1.2 nS; and its next four nearest neighbors with a probability of 0.4 and a conductance of 0.7 nS. Finally, autaptic connections were made from each cell to its own soma, with a randomly distributed peak conductance from a log-normal distribution with a mean of 11 nS, a coefficient of variation of 1 and a synaptic delay of 1 ms [15]. Both classes of GABAergic current had a biexponential decay, with autaptic currents decaying with time constants of 2 and 18 ms (60% fast [15]) and basket cell to basket synapses decaying at 1.4 and 9.3 ms (80% fast [10]). Each GABAergic synapse had a reversal potential of −78 mV [15]. To drive the network to activity, each neuron received a depolarizing injection of current to mimic metabotropic glutamate receptor or kainate receptor activation. This current was drawn randomly from a normally distributed population with a mean of 150–600 pA and a coefficient of variation of 0.1. The current started randomly within the first 50 ms of the simulation and was delivered to the soma. At the start of the simulation, the neurons were instantiated at their resting membrane potential of −68 mV (properties summarized in table 1). For each combination of parameters (inhibitory divergence, gap junction coupling and depolarizing current) the simulation was run 10–15 times.

**Table 1:** Standard parameter and properties for neurons in the model.

Resting membrane potential	Whole-cell Capacitance	Input resistance	E_GABA_	Autaptic Conductance	FS→FS Synaptic Conductance	Action Potential Threshold
−68 mV	59 pF	71 MΩ	−78 mV	11 nS	1 nS	−50 mV

To quantify the level of synchrony in the network, I used the measure χ, which is defined as the square root of the variance of the average membrane potential of every cell in the network divided by the average variance of each cell in the network. χ fluctuates between 0 and 1, 1 being perfectly synchronous (the membrane potential for all cells is equal at all times), 0 being completely random [28]. χ was calculated over the last 200 ms of the simulation. In order to quantify the frequency at which the network was operating, and to give a surrogate measure of network synchrony, the times of all action potentials in the network were captured and binned at 0.5 ms. This histogram was subjected to a fast-fourier transformation (Hanning window, FFT size 256) and the peak frequency and power between 30 and 300 Hz was measured. It is worth noting that χ takes into account subthreshold and suprathreshold membrane synchrony, while the power of the spiking oscillations just observes the synchrony of firing. I chose not to use the population coherence measure κ because it only takes spiking synchrony into account and not subthreshold synchrony. Also, I found any published descriptions of the method used to calculate κ opaque to understanding. Hypothesis testing was conducted using a univariate general linear model with PASW 18.0 (SPSS, Hong Kong). Action potential threshold was defined as the mean voltage when the third time derivative of the voltage signal filtered at 10 kHz stayed positive for more than 10 samples in a row.

In order to model synaptic depression, I imagined that the peak conductance of a synapse at any given time is the product of its resting synaptic conductance and a synaptic resource *R*, which could vary between 1 and 0. At time 0, *R* has a value of 1, and after each action potential *R* is multiplied by a certain constant, *d* (which varies between 0 and 1). *R* then recovers back to 1 via a (multi)exponential process which was defined by the measured time constant for recovery from synaptic depression. This is an attractive model for several reasons. Firstly, it has only one unknown constant, *d*, which can easily be found by fitting the model to recorded data. Secondly, it is computationally inexpensive, as it can be solved by analytical methods, once per action potential. This model is conceptual similar to that of Tsodyks and Markram [29] and Fuhrmann et al., [30]. For autaptic connections, depression was best fit with *d* = 0.3 and recovery fit with three exponentials with decay constants of 10 ms (20%), 56 ms (30%) and 4156 ms (50%) [15]. For the inhibitory connections between basket cells, data was gathered from Kraushaar and Jonas [31], *d* = 0.3 and recovered with a biexponential process with decay constants of 10 ms (40%) and 1970 ms (60%). I found that I needed a very fast recovery process because otherwise the model could not fit the basket cell→basket cell data, hence I chose 10 ms arbitrarily. I then assumed that the biophysics of the recovery process at autaptic synapses was probably very similar to basket cell→basket cell synapses, and hence I included the 10 ms decay constant there as well.

## Results

**Figure 1:**
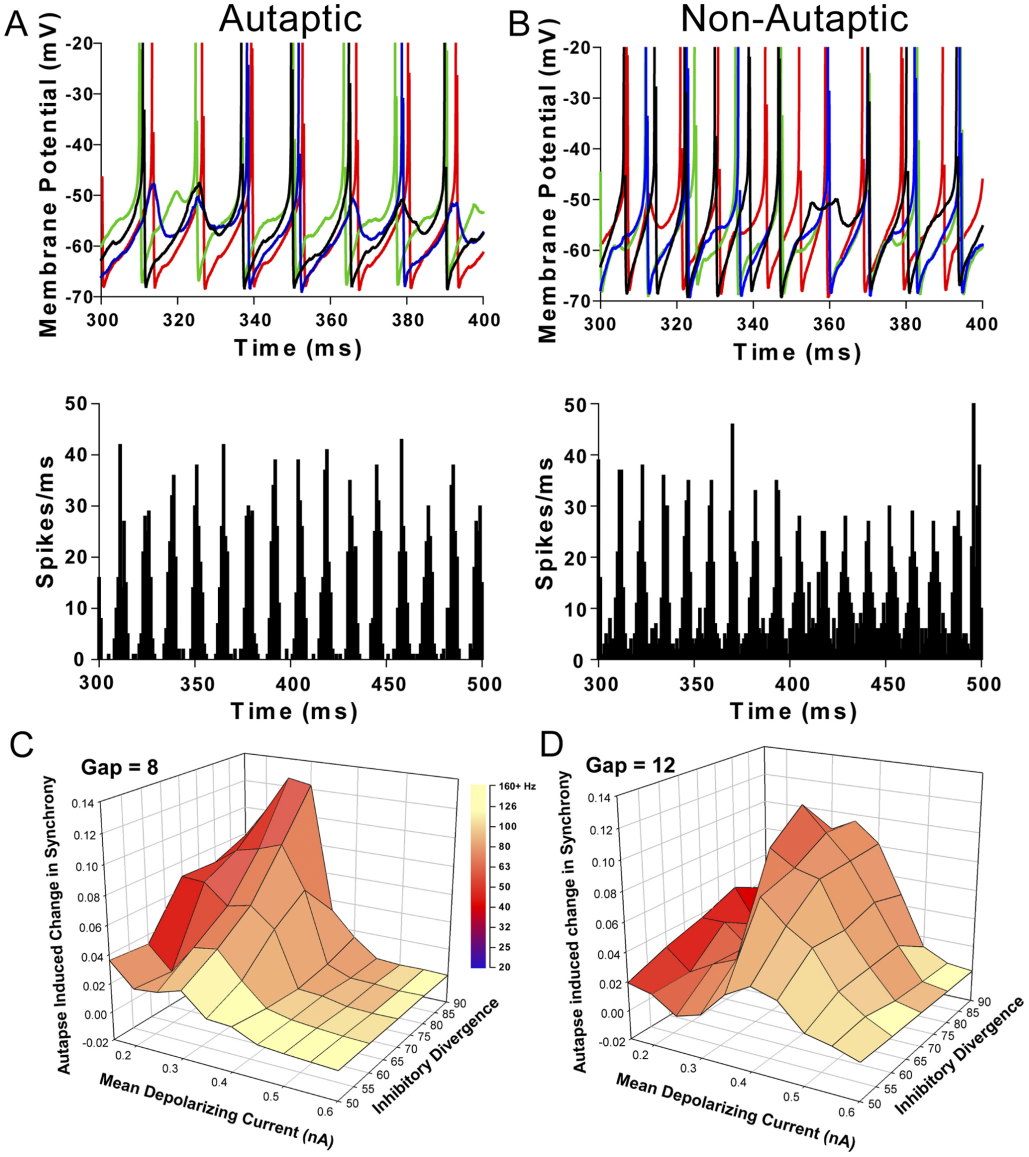
Autapses enhance cell synchrony in a model of gamma oscillations. ***A.*** A representative run showing network behavior when cells are equipped with autapses. The network has a mean depolarizing current of 300: Membrane potential oscillations in 4 cells (50 cells apart) showing synchronous firing and subthreshold membrane potential oscillations. Lower panel: A histogram including the same time period as the upper panel showing action potentials over the last 200 ms of the simulation over all cells. This simulation run has a synchrony score of 0.14. ***B.*** Network behavior in a non-autaptic model. The network has the same properties as *A*, but without the presence of autapses. Upper panel: Membrane potential oscillations in 4 cells (50 cells apart) showing a lower level synchronous firing and subthreshold membrane potential oscillations. Lower panel: A histogram including the same time period as the upper panel showing action potentials over the last 200 ms of the simulation over all cells. This simulation run has a synchrony score of 0.09. ***C*** **.** The change in synchrony produced by the presence of autapses in a network with a gap junctional coupling of 8. The color shows the frequency of the network oscillation ***D.*** When the maximum number of cells each neuron can contact is increased to 12, the area of parameter space where autapses can enhance synchrony is reduced, however the maximum enhancement is unchanged.

All previous models of gamma oscillations have ignored the presence of autaptic connections, and I therefore sought to test whether autapses help to enhance the synchrony of interneuron activity during gamma oscillations. I developed a model of basket cells based on realistic assumptions about the cellular, synaptic and network properties of neocortical interneurons (see Methods). The basic parameters and emergent properties of the cells in the network are outlined in table 1. When the cells in the network were stimulated with randomly sampled depolarizing current (mean  = 150–600 pA, coefficient of variation  = 0.1, initiated randomly over the first 50 ms of the simulation), robust oscillations developed in the 40–120 Hz range (Fig 1AB). Networks with higher degrees of inhibitory divergence (*F*_(3,3193)_  = 165, *P*<0.001) and gap junctional coupling (*F*_(2,3193)_  = 223, *P*<0.001) had the highest degree of synchrony (e.g. an autaptic network with divergence of 80 and gap junctional coupling of 12 at 150 pA χ = 0.16±0.015 frequency  = 40 Hz, vs an autaptic network with divergence of 50 and gap junctional coupling of 8 at 150 pA χ = 0.06±0.009). The presence of autapses allowed the network to produce synchronous (χ>0.01) oscillations over a wider range of depolarizing current amplitudes. For example when the gap junctional coupling was 10, a depolarizing current of 150–450 pA produced synchronous oscillations in an autaptic network while this was restricted to 150–300 pA in a non-autaptic network (See Figure 1CD for an exploration of parameter space where autapses enhanced synchrony). Furthermore, autapses increased the peak synchrony by 50–160% (across different levels of inhibitory divergence, the peak synchrony increased by an average of 0.05, or 100%; F_(1,3193)_  = 1359, *P*<0.001; Fig 1C). No combination of parameters resulted in autapses decreasing synchrony significantly. Analysis of the power of oscillations in spiking rate of the network showed a very similar trend, with the presence of autapses increasing the power of the oscillations in the spiking rates (*F*_(1,3193)_  = 512, *P*<0.001). This was most notably seen when current injection was less than 400 pA.

**Figure 2:**
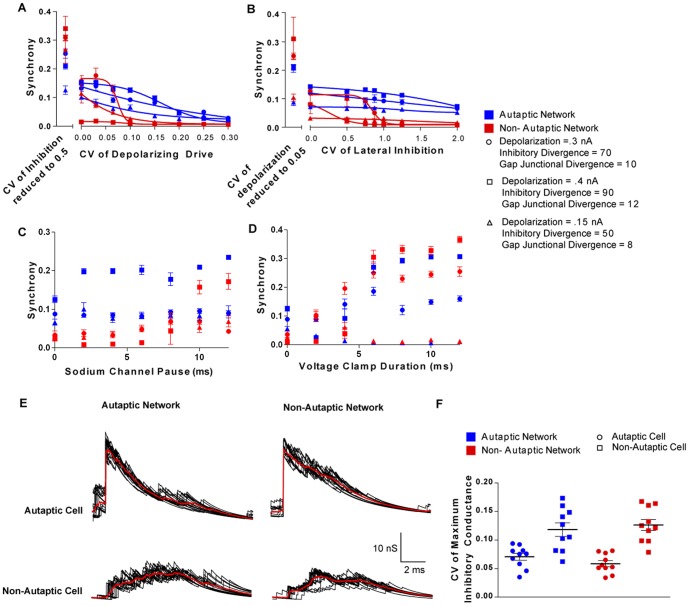
Autapses appear to enhance network synchrony by reducing the effect of variance in depolarizing drive as well as in synaptic IPSCs. ***A.*** Over a range of network parameters, the presence of autapses allows networks to generate synchronous oscillations when the coefficient of variation (CV) of the depolarizing drive is increased. On the other hand, decreasing the CV of the depolarizing drive largely mimics and occludes the effect of autapses. By concomitantly halving the CV of the amplitude of cell to cell inhibitory synapses, autapses no longer enhance synchrony over any condition. ***B.*** Autapses allow networks with a high degree of variance in the amplitude of cell to cell (lateral) inhibitory synapses to produce synchrony oscillations. By reducing the CV of the amplitude of lateral synapses, the effect of autapses in enhancing network synchrony is somewhat reduced, and by concomitantly halving the CV of the depolarizing drive, autapses no longer enhance synchrony over any condition. ***C.*** Autapses do not enhance synchrony by preventing action potential bursts. The model of the voltage gated sodium channel was modified such that its conductance was reduced to zero for a period after the action potential (sodium channel pause). Varying the length of this pause from 2–12 ms had no effect on network synchrony. ***D.*** Autapses no longer enhance synchrony, and in fact decrease it when a dynamic voltage clamp is induced to mimic the effects of autapses. In this model, each cell was subject to a perfect voltage clamped at −78 mV for a variable period of time. When the duration of the clamp is greater than 4 ms, autapses no longer enhance synchrony. ***E.*** Autapses reduce the CV in the post action potential conductance. Over-laid traces time locked to the action potential (the start of each trace) showing the total inhibitory conductance each cell receives. In the autaptic cell-non-autaptic network case, only the cell being recorded from has an autapse, while in the non-autaptic cell-autaptic network only the cell being recorded from is lacking an autapse. This network has a gap junctional coupling of 10, an inhibitory divergence of 70 and a depolarization drive of 0.3 nA. ***F.*** Whether in an autaptic network or not, autapses decrease the CV of the inhibitory conductance. (Effect of autapses in the measured cell on CV: *F*_(1,40)_  = 45 *P*<0.001. Effect of autapses in the network on CV: *F*_(1,40)_  = 0.05 *P* = 0.8. Interaction *F*_(1,40)_  = 1.3 *P* = 0.3). Each panel in *E* is generated by randomly selecting a cell in the network, and measuring all of the post action potential phases during the last 200 ms of the simulation. Each data point in *F* is generated by taking a sample of data like in *E*, and sampling the peak conductance in each cycle, and calculating the CV from that population (i.e. each data point represents one cell, in one trial).

**Figure 3:**
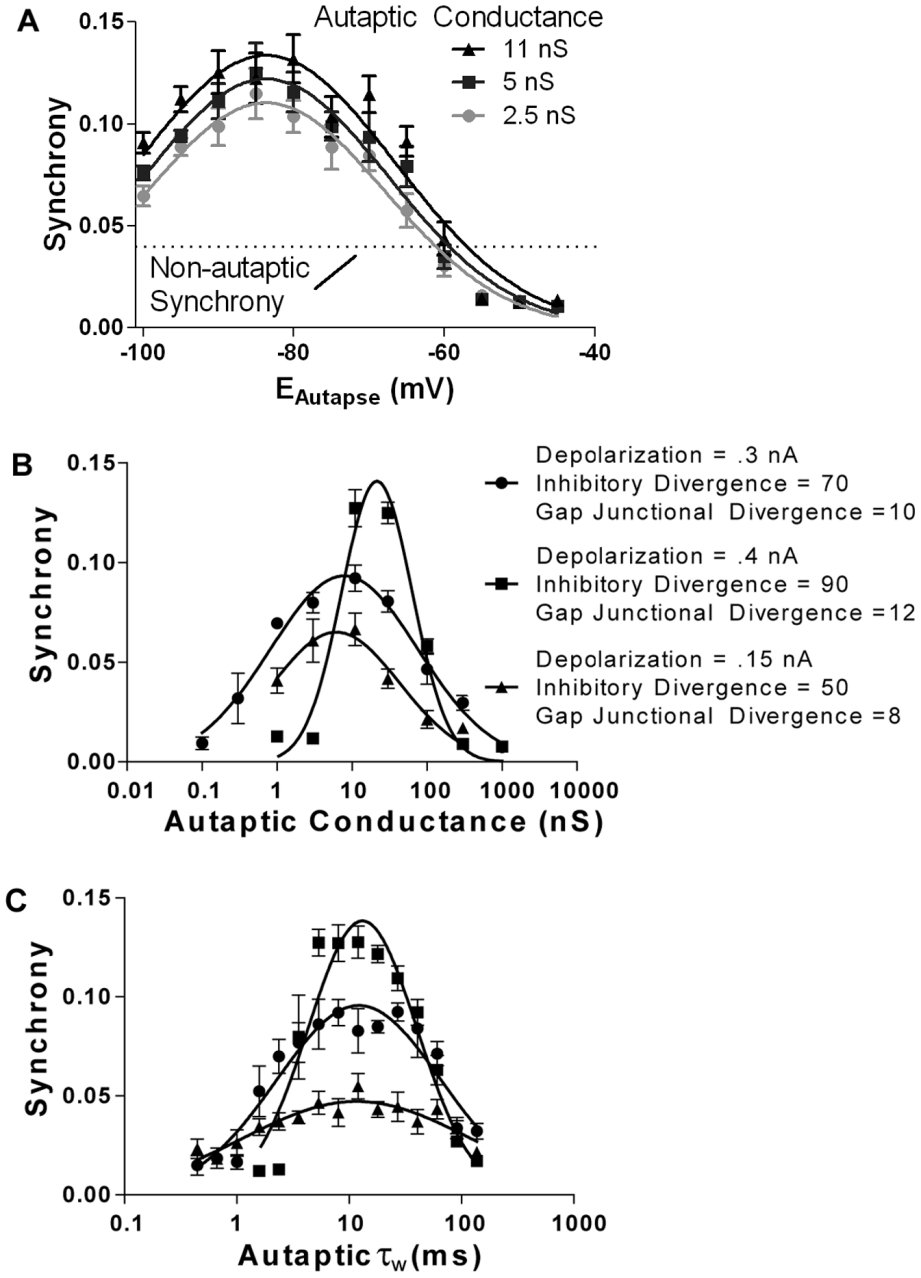
Real autaptic parameters are tuned to those which enhance synchrony. ***A.*** The reversal potential of autaptic currents (but not that of FS→FS synapses) was changed across a simulation with a mean depolarizing current of 150 pA, a gap junctional coupling of 10, and an inhibitory divergence of 70. The reversal potential of autaptic currents need to be between −100 mV and −55 mV to enhance synchrony. ***B.*** As the mean autaptic conductance is varied, network synchrony is altered, peaking at values between 6–21 nS (the peaks of the log-normal regression curves). ***C.*** As the weighted decay constant of the bi-exponential decay is varied (amplitude ratio constant at 0.6), network synchrony is altered, peaking at values between 11.5–13 ms (the peaks of the log-normal regression curves).

I initially hypothesized that autaptic transmission enhanced the synchrony of the network by providing a high conductance shunt that provided a phase where it was very unlikely that a cell would fire an out of phase action potential. However, when I altered the nature of the voltage-gated sodium channel such that its conductance was reduced to zero after the action potential for a period ranging from 1–12 ms, this did not mimic the synchrony enhancing effect of autapses (Fig 2C). This means that autapses do not enhance synchrony simply by silencing cells after they spike. I then suspected that autapses might increase synchrony by providing a high conductance input that reduces the coefficient of variance in the GABAergic conductance during the post action potential phase. Indeed, I found that with most combinations of parameters, autapses no longer enhanced network synchrony when the variance of the size of the lateral synaptic inhibition was reduced to zero. However, in order to make autapses completely redundant, the variance in the magnitude of the depolarizing current as well as the variance in the amplitude of synaptic inhibition needed to be reduced (Fig 2AB). Conversely, this also shows that by increasing the variance of lateral inhibition, the synchrony in an autaptic network is also significantly reduced (*F*_(6,175)_  = 15, *P*<0.001). Furthermore, by introducing a voltage clamp to each cell in the network, which was active for a short period of time (2–12 ms) after each action potential (i.e. following a similar pattern to autaptic transmission), the synchrony enhancing effect of autapses could be mimicked (Fig 2D). Thus it appears that autapses enhance synchrony by normalizing the conductance during the compound IPSC experience by a cell after the action potential. That is to say, by providing a consistent inhibitory conductance the autapse causes the net post action potential conductance to take on a stereotyped form (Fig 2E), with a lower total coefficient of variation (Fig 2F). Indeed, this can be demonstrated by the fact that the presence of an autapse in one cell in an otherwise non-autaptic (and hence less synchronous) network reduces the coefficient of variation of inhibition in that cell (Fig 2EF). These experiments also revealed that autapses allow networks to produce synchronous oscillations over a wider range of variance in the magnitude of the depolarizing current than the classical model (Fig 2A). However, while an autaptic network would oscillate when receiving a depolarizing drive with a coefficient of variation of up to 15–20%, this still does not quite reach the physiological level of excitatory drive recorded in brain slice models of gamma oscillations (approximately 35% see [8]). The notion that autapses increase synchrony by providing a high conductance shunt that normalizes post-action potential inhibitory currents is supported by the fact that by changing the reversal potential of autaptic currents (E_Autapse_) to either more hyperpolarized (<−90 mV) or more depolarized (>−75 mV) decreases the synchrony of the network (Fig 3A). In an autaptic network, maximal synchrony was produced with E_Autapse_  = −80 mV. If autapses increased network synchrony by hyperpolarizing the cell, one would expect their effect to increase as their reversal potential was made more negative, which was not the case. If E_Autapse_ was more depolarized than −60 mV, autapses decreased synchrony to levels less than non-autaptic networks. Thus defining the exact reversal potential of autaptic currents is crucial to the interpretation of this study. While we have previously measured the reversal potential for perisomatic GABA_A_ currents in autaptic neocortical interneurons to be −78 mV [15] this is significantly more hyperpolarized than that measured in other fast-spiking cells in the neocortex (−55 mV [32]) and basket cells in the hippocampus (−52 mV [33]). However, these are not the reversal potential for autaptic currents, but rather general inhibition and basket cell → basket cell synapses respectively. Gramicidin perforated patch recordings from both our lab and other groups have not been able to record an autaptic IPSP even while measuring their ability to inhibit action potentials [14], [15]. As the autaptic conductance is very large, this strongly indicates that the reversal potential for autaptic currents is close to the resting membrane potential, i.e. that it acts via shunting inhibition.

If the presence of basket cell autapses was evolutionarily selected for because of their ability to enhance gamma oscillations, it would seem likely that their native properties would be maximally suited to enhancing network synchrony. By varying autaptic kinetics and conductances, I noted that autapses were the most effective at enhancing network synchrony when they decayed with a τ_w_ of 11.5–13 ms, and had a conductance of 6–21 nS, close to the measured values of 8 ms and 10.7 nS (Fig 3BC).

**Figure 4:**
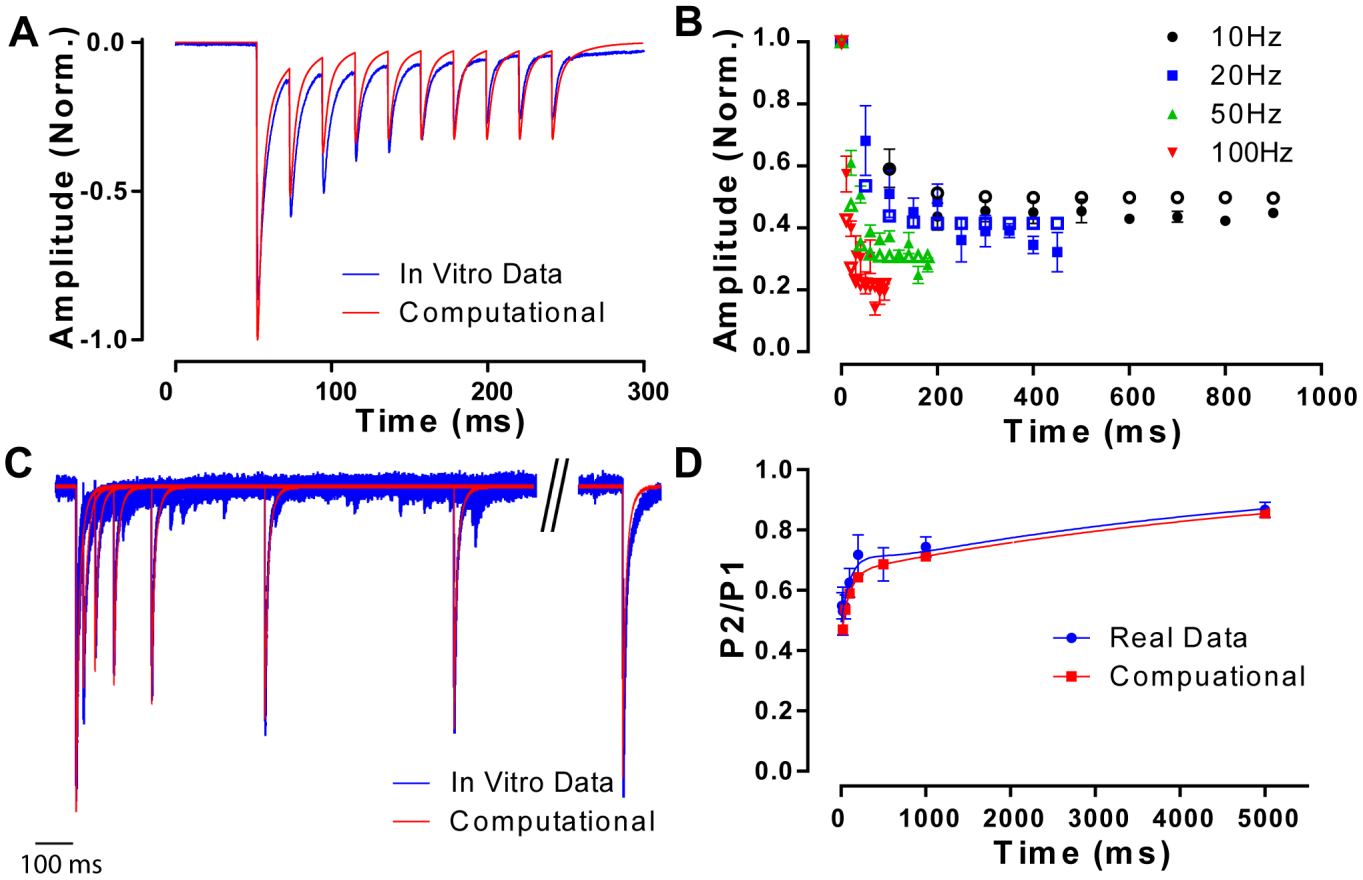
A simple model of paired pulse depression accurately modeled synaptic plasticity as seen in vitro. ***A.*** A recorded autaptic burst at 50(blue) showing paired pulse depression (data taken from [15]). The computation model (red) is a close fit. ***B.*** Summary statistics showing paired pulse depression at autaptic connections in response to trains at several different stimulus intensities during in vitro recording (filled symbols) and during a computational model (open symbols). ***C.*** An *in vitro* recording of the recovery from synaptic depression (blue) fits closely with the recovery as generated by the model (red). ***D.*** Measurements of the computational model perfectly recreate the recovery from synaptic depression. Results are reported as the amplitude of the second synaptic event (P2) as divided by the amplitude of the first synaptic event (P1).

**Figure 5:**
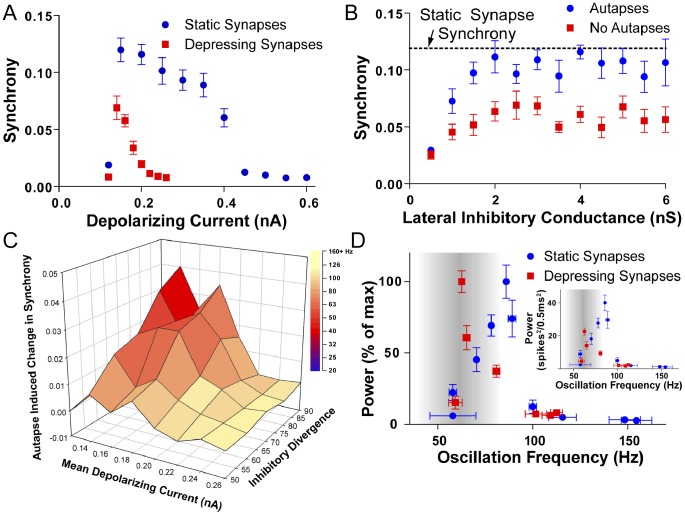
The effect of depressing synapses on the WB model of gamma oscillations. ***A.*** Including depressing synapses in the WB model reduces the peak synchrony as well as the range of depolarizing current that can produce synchronous oscillations. ***B.*** The average lateral inhibitory conductance needs to be doubled before networks with depressing synapses can produce oscillations as synchronous non-depressing networks. The dotted line represents the mean synchrony of the network under identical conditions apart from using non-depressing synapses. ***C.*** Even in a network with depressing synapses, autapses enhance synchrony. ***D.*** Depressing synapses help constrain oscillations to the gamma band. During various levels of depolarizing drive, the network produces oscillations across different frequencies (larger depolarization generates higher frequencies). By analyzing the peak spectral power of spike times of the network, it can be seen that networks with depressing synapses are tuned to oscillate at 60 Hz range, while non-depressing networks are tuned to 90 Hz. Inset, a non-normalized representation of the power of the oscillation against frequency. *A*, *B* and D were run with inhibitory divergence of 70, gap junctional coupling of 10 and a depolarizing current of 0.15 nA.

I have shown that FS cell autapses are depressing, and it is well established that the synapses between FS cells are also depressing e.g. [34]. As mentioned earlier, the WB model is the most cited model of gamma oscillations, and has been the basis of numerous studies, however, all implementations of the WB model either do not include synaptic depression e.g. [10], [33], or simply reduce synaptic conductances to model synaptic depression at “steady state” (which of course, is not reached unless the neuron fires at a constant frequency) e.g. [35]. Thus, I sought to investigate how robust the WB model was if synapses were accurately modeled as depressing, and whether autapses still enhanced network synchrony if they had appropriate short term plasticity. I formulated a simple model of synaptic depression where the output of a synapse is the product of its resting conductance and a synaptic resource (see methods for details). Our simple model seemed to accurately recreate short term plasticity, mimicking both the time course of a single event (Fig 4A), the frequency dependence of depression (Fig 4B) and the recovery from depression (Fig 4CD). Therefore, I used it in the computational network to test the effects of dynamic synapses in the WB model. Including depressing synapses in the WB model reduced the network synchrony for all combinations of parameters (Fig 5). Furthermore, synchronous oscillations could only be generated over a smaller range of depolarizing currents (0.12–0.2 nA vs 0.12–0.4 nA). This is not surprising, as during the trains of activity seen during oscillations, lateral inhibitory synapses would depress down to about 25% of the resting conductance, and lowered lateral inhibition is known to reduce the synchrony in gamma oscillations model [8]. By doubling the mean lateral inhibitory conductance I generated network activity in models with depressing synapses as synchronous as those with static synapses (Fig 5B). However, even with depressing synapses, autapses still enhanced network synchrony (Fig 5BC). Interestingly, the fact that depressing synapses restricted the range of depolarizing currents that could produce synchronous oscillations had an unexpected side effect. Over the range of currents that could produce oscillations, networks with static synapses could produce oscillations in the 40–120 Hz range (higher frequencies can be reached at higher levels of inhibitory divergence). However, when depressing synapses are included, networks oscillate only in a 60–80 Hz range. Thus depressing synapses act to keep oscillations at a restricted frequency, at the expense of absolute synchrony.

## Discussion

Our network simulation indicates that autapses enhance the synchrony of basket cell networks when they oscillate in the gamma range (40–80 Hz). Furthermore, it appears that the kinetics, the size of the conductance and the reversal potential of autaptic connections *in vitro* are close to the maxima for supporting synchronous network oscillations *in silico*. It seems that autapses increase network synchrony by normalizing the variability in cell to cell inhibition in the post-action potential phase of the cycle. This conclusion was supported by the observation that the effect of autapses could not be replicated by preventing neurons from firing directly after an action potential (i.e. the effect of autapses was not due simply to inhibiting cells firing). However, the effect of autapses were mimicked and occluded by brief (>6 ms) voltage clamps (with a command potential equal to the reversal potential for inhibition) applied to the soma of neurons directly after the action potential. It is important to note that 2–4 ms long voltage clamps did not achieve this, indicating that the effect of the voltage clamp was not simply due to it transiently pulling the neuron down to a hyperpolarized membrane potential. Similarly, by modifying the reversal potential for autaptic conductances, it was seen that autapses are the most efficient at enhancing network synchrony when the reversal potential is near −80 mV, i.e. that they provide shunting inhibition. This further supports the notion that autapses increase network synchrony not by hyperpolarizing basket cells, nor by transiently silencing them. The presence of autapses allows networks to continue to produce synchronous oscillations when the variability in the excitatory drive is high, and approaching physiological levels, something that causes previous models of gamma oscillations to collapse as reviewed by [8]. This result also stresses the importance of accurately modeling conductances which generate the AHP (rather than the single potassium conductance that is used in the WB model) in future models of gamma oscillations. I also modified the WB model to include accurately depressing synapses for the first time. This significantly reduced the synchrony of the network, but also filtered the output, such that the network would only produce synchronous oscillations in the gamma frequencies.

When one considers the potential role of synchronous gamma oscillations in the generation of consciousness [36], a likely benefit of a network of neurons that can only generate oscillations at a restricted frequency is that it means synchrony can be achieved more simply. Rather than the network having to lock both the phase and the frequency to achieve synchronous oscillations across disparate parts of the neocortex, it simply needs keep the two regions in phase.
